# Corrosion of Stainless Steel in Simulated Nuclear Reactor Primary Coolant—Experiments and Modeling

**DOI:** 10.3390/ma17051148

**Published:** 2024-03-01

**Authors:** Martin Bojinov, Iva Betova, Vasil Karastoyanov, Georgi Avdeev

**Affiliations:** 1 Department of Physical Chemistry, University of Chemical Technology and Metallurgy, 1756 Sofia, Bulgaria; vasko_kar@uctm.edu; 2 Institute of Electrochemistry and Energy Systems, Bulgarian Academy of Sciences, 1113 Sofia, Bulgaria; i.betova@iees.bas.bg; 3 Institute of Physical Chemistry, Bulgarian Academy of Sciences, 1113 Sofia, Bulgaria; g_avdeev@ipc.bas.bg

**Keywords:** 316L stainless steel, primary water chemistry, electrochemical impedance spectroscopy, oxide growth, corrosion release, mixed-conduction model

## Abstract

In the present paper, the effect of the evolution of primary water chemistry during power operation on the corrosion rate and conduction mechanism of oxide films on stainless steel is studied by in situ impedance spectroscopy at 300 °C/9 MPa during 1-week exposure periods in an autoclave connected to a recirculation loop. At the end of the exposure period, the samples were anodically polarized in a wide range of potentials to evaluate the stability of the passive oxide. Separate samples of the same steel were simultaneously exposed to the coolant and subsequently analyzed by glow discharge optical emission spectroscopy (GDOES) in order to estimate the thickness and the in-depth composition of the formed oxides. Impedance data were quantitatively interpreted using the mixed-conduction model for oxide films (MCM) to estimate the rates of metal oxidation at the alloy/oxide interface, oxide dissolution and restructuring at the film/coolant interface, and ion transport in the protective corrosion layer.

## 1. Introduction

According to the International Energy Agency World Energy Outlook 2022, nuclear energy, with more than 400 reactors in operation in more than 30 countries, was the second low-carbon energy source in the world in 2021, comprising 9.8% of electricity production. Most of the reactors in use are pressurized water reactors (PWRs) consisting of a primary (energy-generating) loop and a secondary (electricity-generating) loop. Currently, there are two such reactors in operation in Bulgaria, with extensive plans to build two more in the coming decade.

The internal components of light water reactors fabricated from austenitic stainless steel are exposed to intense neutron irradiation, mechanical and thermal stresses, and the corrosive action of the coolant water. This exposure leads to several degradation mechanisms, limiting the lifetime of the internals. All corrosion modes are significantly influenced by the primary coolant chemistry. Boron–lithium buffer chemistry is used in most primary water of pressurized water reactors (PWRs) to achieve a moderate alkaline pH at operating temperatures (6.9–7.3), at which Fe and Ni solubility is minimized [1] and, thus, corrosion and corrosion product release rates are reduced. Recently, concerns were raised about the supply of Li, especially isotopically enriched ^7^Li, because of the uncertain future availability of Li and the high cost of producing the enriched material [2]. Considering the success of using KOH for pH control in water–water energy reactors (WWERs) [3], PWRs are now also considering replacing LiOH with KOH [4,5,6,7,8,9,10,11,12,13,14]. However, differences in the design and materials used in PWRs highlight the necessity of studying the compatibility of KOH with structural materials of PWRs. An analysis of the experience gained for more than 40 years of operation of NPPs equipped with WWER reactors with the 12-month fuel cycle and operation of power units at the nominal level of power shows that by now, no real cases with the occurrence of axial offset anomalies have been recorded in power units based on WWER reactors. This can be attributed to the following: first, potassium hydroxide, the solubility of which is almost 100 times higher than that of lithium borates, was used as a pH-correcting agent [15]. Thus, the possibility of local crystallization of boron in the pores of deposited corrosion products in the reactor core can be excluded under the same conditions. Second, alloys with a high content of nickel are not used in the primary coolant circuit; therefore, the content of nickel in the coolant is too low for a considerable amount of nickel ferrite to be produced on the fuel rod cladding [16].

The use of KOH for PWR primary coolant pH control appears to be a promising and economical alternative to LiOH; however, several significant knowledge gaps remain in terms of compatibility of materials and chemistry issues. One key issue concerns the fact that boron undergoes a nuclear reaction, generating lithium, when exposed to the neutron fluxes present in PWRs and WWERs. As a result, WWERs operate with both potassium and lithium from boron conversion in the primary system, while PWRs only operate with lithium. The presence of both cations complicates resin management and pH_T_ control [5,6,7,8]. In this context, it is important to note that the general corrosion rate, the rate of initiation of stress corrosion cracking (SCC), and the crack growth rates of PWR materials all have some dependence on pH_T_ [9,10,11,12,13,14]. Some studies have been completed that isolate the effects of lithium and boron from the effects of pH_T_ on corrosion, but there are major gaps in the present understanding of how these rates would change with high concentrations of potassium in the reactor coolant system.

The general aim of our project is to quantify the oxide formation and corrosion release rates of 316L stainless steel and Alloy 690 (typical PWR internal and tubing materials) in simulated WWER reactor primary coolant at different stages of operation. In the present paper, the focus is on the corrosion behavior of 316L stainless steel as a representative PWR internal. For this purpose, in situ chrono-potentiometric (corrosion potential vs. time) and electrochemical impedance spectroscopic (EIS) measurements are performed. The phase and in-depth elemental composition of the oxides are characterized via glow discharge optical emission spectroscopy (GDOES) and grazing incidence X-ray diffraction (GI-XRD). Kinetic and transport parameters of oxide growth and metal release are estimated through quantitative comparison of the EIS data with the equations of the mixed-conduction model for oxide films (MCM). Based on the water chemistry dependence of these parameters, conclusions regarding the compatibility of LiOH and KOH water chemistries for PWRs are drawn.

## 2. Materials and Methods

**Figure 1:**
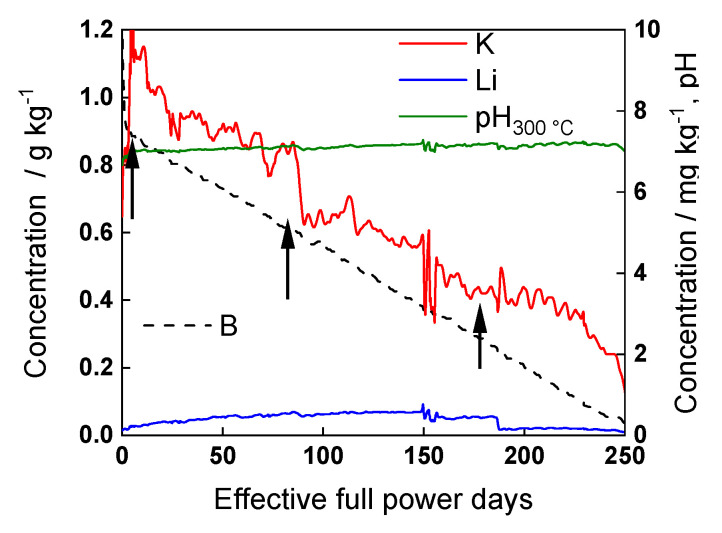
Typical WWER primary water chemistry during operation. Simulated coolants chosen for the measurements are marked with arrows.

The composition of the studied AISI 316L stainless steel (both nominal and analyzed by GDOES) is presented in Table 1. Prior to exposure, the samples were mechanically polished to a mirror finish with emery paper and diamond paste. Experiments were performed at 300 ± 1 °C, with a pressure of 9.5 ± 0.1 MPa, in an autoclave made of the same steel connected to a re-circulation loop, with conductivity controlled using a 912 apparatus (Metrohm, Herisau, Switzerland), pH via a 781 pH/ion-meter (Metrohm, Herisau, Switzerland), and dissolved oxygen content of the coolant using an amperometric micro-sensor (AMT Analysenmesstechnik GmbH, Rostock, Germany). WWER primary coolant at three stages of operation—beginning-of-cycle (BOC), mid-cycle (MOC) and end-of-cycle (EOC)—was used (marked with arrows in Figure 1) and compared to a nominal PWR coolant (1 g kg^−1^ B, 1 mg kg^−1^ Li) with identical pH at the measurement temperature. The reference nominal PWR primary chemistry was deliberately chosen to correspond to the maximum amount of Li formed during a WWER campaign. No NH_3_ was added to minimize the effect of hydrogen reactions on the electrochemical response. In a typical experiment, after mounting the electrodes and filling the loop with coolant, the system was heated to 80 °C and purged with N_2_ (99.999%) for 16 h. The residual dissolved oxygen concentration after this procedure was below 0.31 µmol kg^−1^. After reaching this value, the temperature gradually increased, with the target value of 300 ± 1 °C being reached within 2–2.5 h at a pressure of 9.5 ± 0.01 MPa.

The corrosion potential and impedance spectra were continuously measured during 1-week exposure periods, followed by anodic polarization of the samples in a large range of potentials to investigate the stability of the passive oxide. A Pt sheet (99.9%) counter electrode and a Pd (99.9%) sheet, cathodically polarized with a current of 10–30 µA vs. an additional Pt to approximate the reversible hydrogen electrode (RHE), completed the 3-electrode setup. All the potentials were recalculated to the standard hydrogen electrode (SHE) scale. A 10,030 Compactstat (Ivium, Eindhoven, The Netherlands) equipped with a frequency response analyzer and operating in a floating mode was used in a frequency range from 11 kHz to 0.1 mHz, with an ac amplitude of 50 mV (rms). The linearity of the impedance spectra was verified by measuring with amplitudes from 20 to 60 mV, whereas causality was checked via compatibility with Kramers–Kronig transforms using the so-called measurement model and associated software [17]. All the experiments were repeated at least three times, and the reproducibility was better than ±1% per impedance magnitude and ±3° by phase angle.

Separate samples for ex situ analysis were exposed in similar conditions. In-depth elemental profiles of the samples after exposure were obtained using GDOES over an area of 5 mm^2^ with a GDA750 analyzer (Spectruma Analytik, Hof, Germany) equipped with a polychromator (focal length 750 mm and grating of 2400 channels/mm). Typical operating parameters were a primary voltage of 950 V, a current of 9 mA, and a pressure of 3 hPa. Calibration was based on certified reference materials chosen to cover the elements present in a wide range of stainless steels in the relevant concentration ranges. Conventional and grazing incidence X-ray diffraction was performed with a PANalytical Empyrean diffractometer (Malvern Panalytical Ltd., London, UK).

## 3. Results

### 3.1. Corrosion Potential with Time

**Figure 2:**
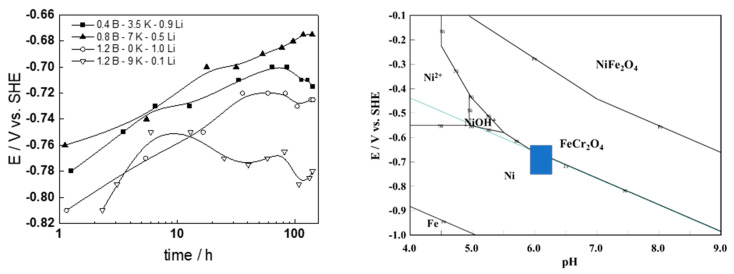
Corrosion potential vs. time, depending on water chemistry (**left**) and the overall potential range, situated in an E-pH diagram of the system Fe-Cr-Ni-H_2_O at 300 °C/9 MPa (**right**).

Corrosion potential increases logarithmically with time, indicating passivation (Figure 2, left). Some fluctuations were observed in beginning-of-cycle (BOC) WWER chemistry. Values after the 1-week oxidation period, situated in the E-pH_300 °C_ diagram of Fe-Cr-Ni-H_2_O at 300 °C/9 MPa (Figure 2, right), indicate that the stable corrosion products were most probably chromite with some metallic Ni incorporated into it.

### 3.2. Current vs. Potential Curves

**Figure 3:**
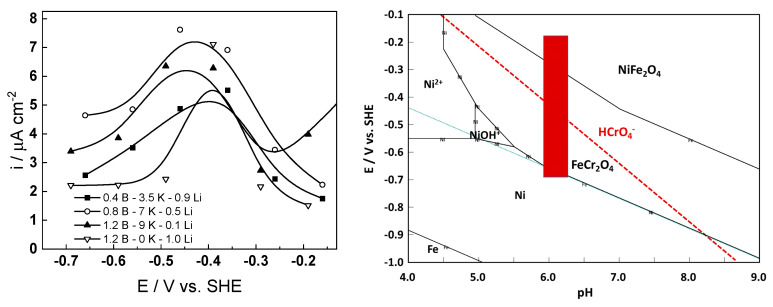
Current vs. potential curves (**left**) and the corresponding potential range situated in the E-pH diagram (**right**).

A current increase was observed in the middle of the studied range of potentials (Figure 3, left), indicating possible transpassive oxidation and secondary passivation. This conclusion was corroborated by the E-pH diagram, in which an intersection of the stability line of HCrO_4_^−^ with a superimposed potential range coincides with the current increase (Figure 3, right).

### 3.3. Electrochemical Impedance Spectroscopy

**Figure 4:**
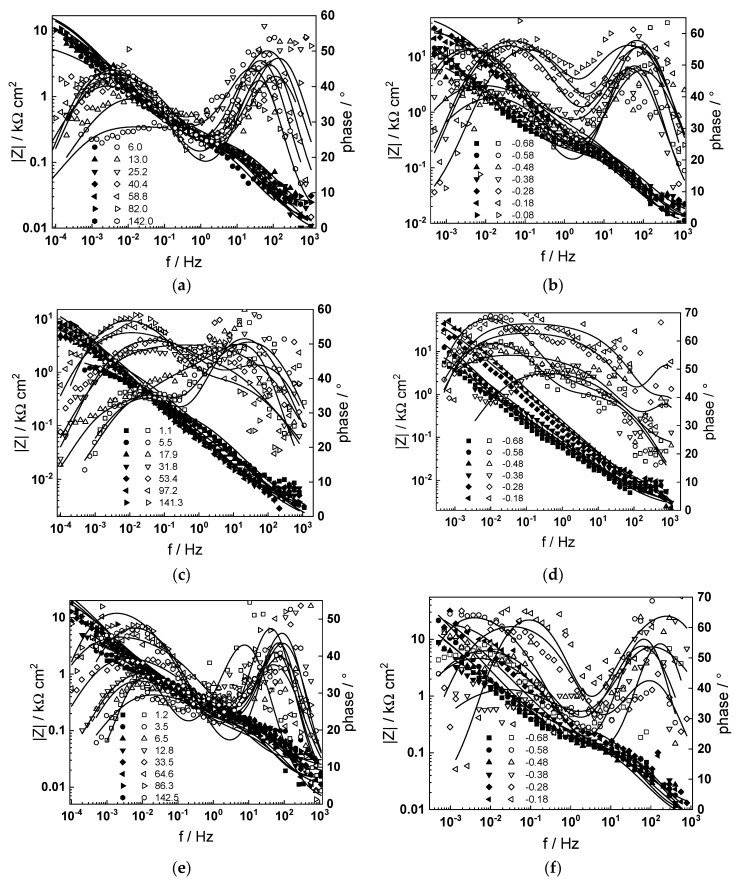

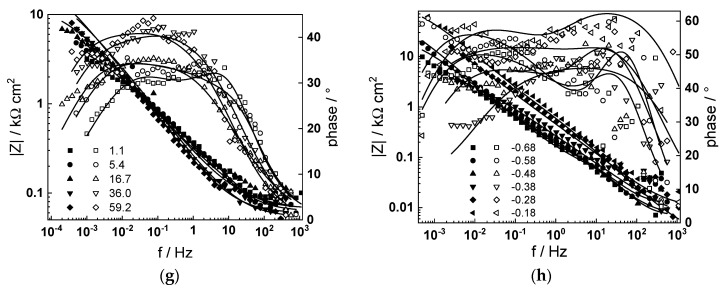
Impedance spectra at different exposure times (**a**,**c**,**e**,**g**) and under anodic polarization (**b**,**d**,**f**,**g**) in WWER BOC (**a**,**b**), WWER MOC (**c**,**d**), WWER EOC (**e**,**f**), and nominal PWR (**g**,**h**) coolants. Left axis—impedance magnitude; right axis—phase shift vs. frequency. Points—experimental values; lines—best-fit calculation.

Electrochemical impedance spectra during exposure and subsequent anodic polarization of the material in the selected primary coolant are collected in Figure 4. Inspection of the spectra reveals that impedance magnitude at f→0 (a measure of the polarization resistance, i.e., the inverse of the corrosion rate) increased slowly with time up to 90–100 h, indicating growth of the passive oxide. For longer durations, a small decrease of |Z|_f→0_ is usually observed, followed by a stabilization. Some of the experiments were carried out for up to 1 month of exposure, and no further evolution of open-circuit potential or impedance was observed. Thus, it was concluded that the system had entered a pseudo-steady state after 1 week of exposure.

Using a distribution of relaxation times method and associated software [18], four time constants were detected in the spectra, corresponding to electronic properties of a barrier sublayer, charge transfer at the oxide/coolant interface, and diffusion–migration of two types of defects through the barrier oxide.

Concerning the dependence of impedance on potential during anodic polarization, it can be stated that the impedance magnitude increased significantly for potentials above the current peak; thus, the secondary passive oxide remained corrosion-resistant. No extra time constants were detected in the entire studied range of potentials, implying no significant changes in the oxidation mechanism.

### 3.4. Thickness and Elemental Composition of the Oxides

**Figure 5:**
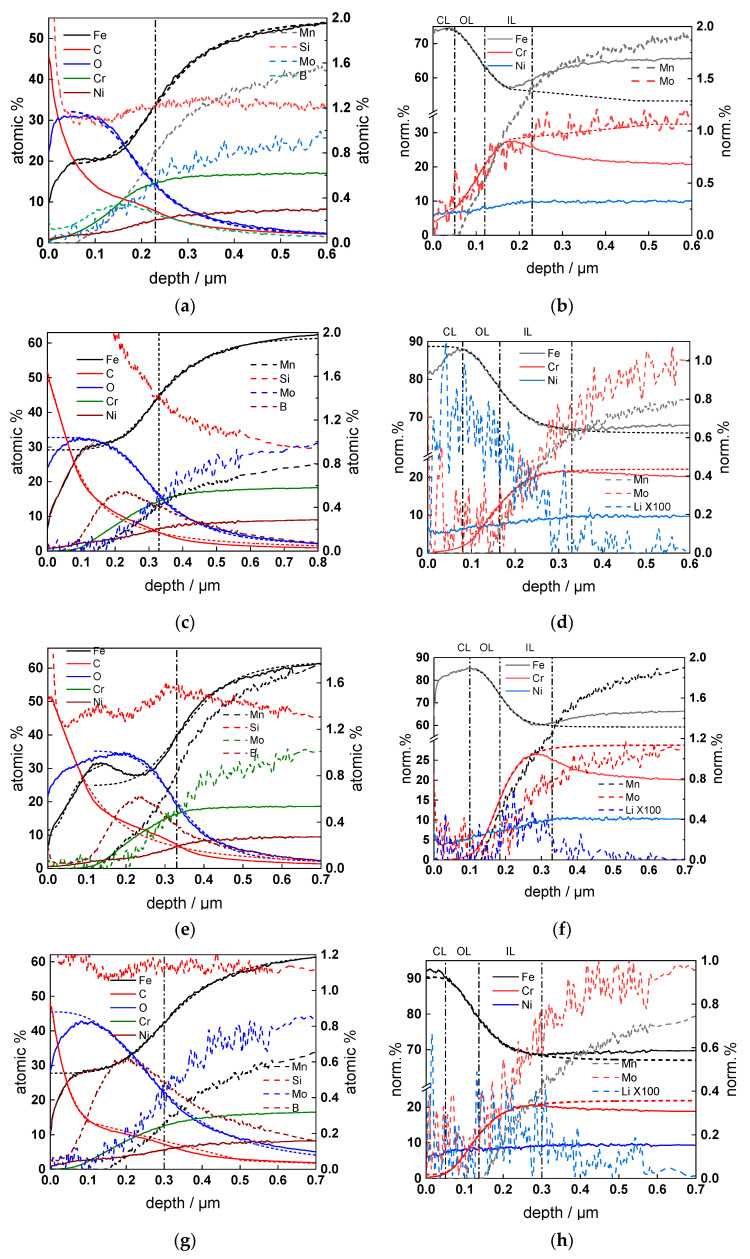
GDOES depth profiles of the elemental composition of oxides formed on AISI 316L in WWER BOC (**a**,**b**), WWER MOC (**c**,**d**), WWER EOC (**e**,**f**), and nominal PWR (**g**,**h**) coolants. Atomic % vs. depth (**a**,**c**,**e**,**g**) and normalized cation composition (**b**,**d**,**f**,**h**) are shown. CL—contamination layer; OL—outer layer; IL—inner layer.

GDOES depth profiles of the oxides after 168 h of exposure to the respective coolants are collected in Figure 5. A bi-layer structure was detected, in agreement with the general expectations for such oxides. The estimated values of inner and outer layer thicknesses are listed in Table 2. The inner layer was slightly enriched in Cr, whereas the outer layer was Fe-rich with some Ni and Cr. Mo and Mn were depleted in the oxide, indicating preferential oxidation and dissolution of these constituents. B presented a maximum between the inner and outer layers, whereas Li was found in small quantities in the outer layer. K was also detected in the outer layer, but was difficult to quantify due to a lack of appropriate standards. The oxide thickness in nominal PWR was comparable to that of EOC WWER. B enrichment between inner and outer layers was also similar, whereas Cr enrichment in PWR was found to be lower than in WWER coolants.

### 3.5. Phase Composition of the Oxide Layer

**Figure 6:**
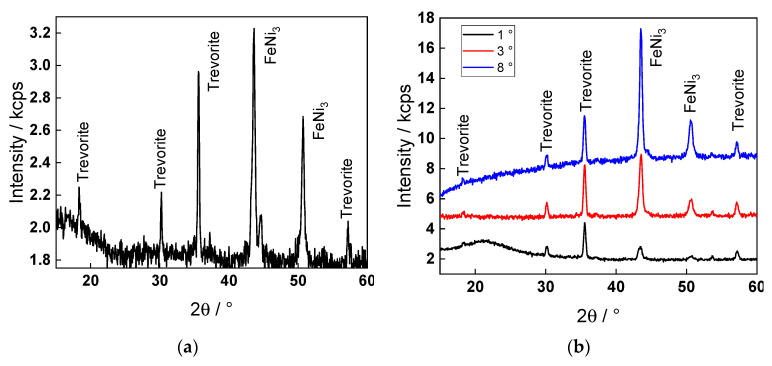
Conventional (**a**) and grazing incidence (**b**) X-ray diffractograms of a WWER BOC sample.

A conventional X-ray diffractogram of the oxide formed in WWER BOC for 168 h is shown in Figure 6a, indicating the presence of nickel ferrite (trevorite, NiFe_2_O_4_) as well as an intermetallic phase close to tetrataenite (FeNi_3_). Measurements with grazing incidence reveal the formation of an underlying phase with a lattice parameter close to chromite (Table 3). The amount of metallic phase increased with the increase in the angle of incidence. Using the calculated unit cell parameters, the composition of the separate phases could be inferred. The lattice constants of (Fe,Cr)(Fe,Cr)_2_O_4_ and (Ni,Fe)(Ni,Fe)_2_O_4_ compositions with various A:B metal ratios were similar enough to those of the pure FeCr_2_O_4_ and NiFe_2_O_4_ spinels. Thus, it can be concluded that the inner/outer layer phases consisted of spinels and inverse spinels of Ni(II)/Fe(III) and Fe(II)/Cr(III) pairs with similar structures to FeCr_2_O_4_ and NiFe_2_O_4_. The formation of a solid solution between the spinel and inverse spinel was rejected based on literature data on oxides formed in similar conditions.

In summary, the in situ electrochemical data indicate a complex oxidation/corrosion mechanism, with both interfacial reactions and transport in the growing oxide playing a role in it. The ex situ characterization points to the formation of a bilayer two-phase oxide and an intermetallic phase, which is in good agreement with thermodynamic predictions. An attempt to rationalize these findings using a kinetic model is made in the next section.

## 4. Discussion

### 4.1. Physical Model

**Figure 7:**
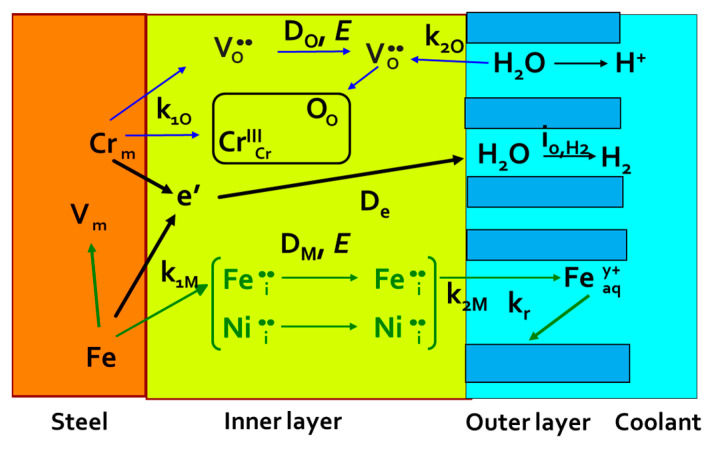
A simplified scheme of the mixed-conduction model for stainless steels [19].

Based on our previous experience with the corrosion behavior of stainless steels in high-temperature water environments [19], the mixed-conduction model for oxide films (MCM) was chosen to interpret the experimental data. A simplified scheme of the model is shown in Figure 7. It features two parallel reaction sequences: inner layer growth by transport of oxygen via the vacancy mechanism and metal dissolution through the oxide (corrosion release and outer layer growth) mediated by the transport of interstitial cations through the inner layer. Both sequences comprised interfacial reactions involving the generation and annihilation of point defects and transport of those defects in the inner layer by diffusion–migration. Electrons from the oxidation reactions were also transported from the inner to the outer interface, reducing water to evolve hydrogen and ensure the continuation of the corrosion reaction. As the protective oxide formed on stainless steel was nano-crystalline, most of the ion transport occurred along the grain boundaries. In that respect, it could be approximated as a homogeneous medium, and unidimensional solutions to transport equations were used. In addition, recent detailed structural investigations of oxides formed on 316 stainless steel fabricated by different methods in PWR medium by high-resolution TEM [20] revealed the homogeneity of the barrier layer in contrast to the outer-layer crystallites and the localized structure of the transition layer between the barrier film and the bulk alloy.

### 4.2. Main Equations for the Impedance Response

**Figure 8:**
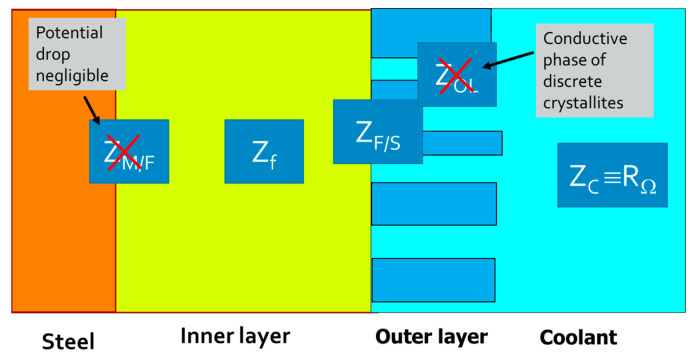
Assumptions made for the derivation of the transfer function.

The assumptions made to derive the transfer function of the impedance are illustrated in Figure 8. The impedance of the outer layer was neglected, since it was not continuous, but consisted of discrete crystallites of a conducting phase on top of the inner layer [20]. On the other hand, the impedance at the steel/inner layer interface was neglected since, at steady-state, the potential drop at this interface was negligible compared to that in the inner layer and at the inner layer/coolant interface [19]. Thus, the overall impedance of the system is the sum of the ohmic resistance of the coolant *R*_Ω_, the impedance of transport of defects in the inner layer *Z_f_*, and the impedance at the inner layer coolant (film/solution) interface *Z_F/S_*:

(1) $$Z=R_{\Omega }+Z_{f}+Z_{F/S}$$

In turn, the interfacial impedance can be expressed as that of a single-step reaction featuring a charge transfer resistance *R_F/S_* and a capacitance *C_F/S_* (generalized as a constant phase element to account for the geometrical and/or energetical heterogeneity at the interface):

(2) $$Z_{F/S}=\frac{1}{R_{F/S}^{-1}+j\omega C_{F/S}}$$

The impedance of the inner layer is a parallel combination of an impedance expressing the electronic properties of the semiconducting phase in the layer *Z_e_* and the transport of ionic point defects (oxygen vacancies and interstitial cations) through it (*Z_i_*):

(3) $$Z_{f}={\left(Z_{e}^{-1}+Z_{i}^{-1}\right)}^{-1}$$

The explicit expressions for these impedances read as

(4) $$Z_{e}\approx \frac{RT}{2j\omega F\vec{E}LC_{sc}}\ln\frac{\left[1+j\omega \frac{RT}{F^{2}D_{e}}\frac{\left(k_{2O}+k_{2M}\right)}{\left(k_{1O}+k_{1M}\right)}\varepsilon \varepsilon_{0}\exp\left(2\frac{F\vec{E}}{RT}L\right)\right]}{1+j\omega \frac{RT}{F^{2}D_{e}}\frac{\left(k_{2O}+k_{2M}\right)}{\left(k_{1O}+k_{1M}\right)}\varepsilon \varepsilon_{0}}$$

(5) $$\begin{array}{l} Z_{i}=\frac{RT}{4F^{2}(1-\alpha )\left\{k_{1O}\left[1+\sqrt{1+\frac{j\omega {\left(RT\right)}^{2}}{{\left(F\vec{E}\right)}^{2}D_{O}}}\right]+k_{1M}\left[1+\sqrt{1+\frac{j\omega {\left(RT\right)}^{2}}{{\left(F\vec{E}\right)}^{2}D_{M}}}\right]\right\}} \end{array}$$

In the above equations, $\vec{E}$ is the field strength in the inner layer; *C_sc_* is the capacitance of the semiconducting phase in that layer; *D_e_*, *D_O_*, and *D_M_* are the diffusion coefficients of electrons, oxygen, and metal cations; and *k*_1*O*_, *k*_1*M*_, *k*_2*O*_, and *k*_2*M*_ are the rate constants at the steel/inner layer and inner layer/coolant interface that are assumed to obey an exponential dependence on applied potential:

(6) $$k_{1M}={k_{1M}}^{0}e^{\frac{3\alpha_{1M}\left(1-\alpha \right)F}{RT}E},\;k_{1O}={k_{1O}}^{0}e^{\frac{3\alpha_{1O}\left(1-\alpha \right)F}{RT}E},\;k_{2M}={k_{2M}}^{0}e^{\frac{\alpha_{2M}\alpha F}{RT}E},\;k_{2O}={k_{2O}}^{0}e^{\frac{\alpha_{2M}\alpha F}{RT}E}$$

In these expressions, *k*_1*O*_^0^, *k*_1*M*_^0^, *k*_2*O*_^0^, and *k*_2*M*_^0^ are the rate constants at *E* = 0; α_1*O*_, α _1*M*_, α_2*O*_, and α_2*M*_ are the respective transfer coefficients; and α is the part of the applied potential consumed at the inner layer/coolant interface. The inner layer thickness-time dependence is given as

(7) $$L=L_{t=0}+\frac{1}{b}\ln\left[1+V_{m,\mathrm{MO}}k_{1O}be^{-bL_{t=0}}t\right],\;b=\frac{3\alpha_{1O}F\vec{E}}{RT}$$

### 4.3. Parameterization of the Model

Parameterization of the model was performed via complex non-linear least squares fitting of the experimental impedance data, depending on the time of exposure and applied potential, using the Levenberg–Marquardt algorithm implemented in an Origin Pro platform. To ensure the statistical viability of the obtained estimates, statistical weighting of data sets was used, and parameters with a mutual dependence higher than 0.67 were not fitted simultaneously. The inner layer thickness at 168 h was taken as equal to that estimated from the GDOES depth profiles of separate samples exposed simultaneously in similar conditions. Impedance spectra depending on potential were first fitted individually. The set of parameters that was obtained was averaged, and a global fit of all the impedance spectra was performed, with potential as an additional variable.

The best-fit results are shown in Figure 4 with solid lines. They demonstrate that both the magnitude and frequency distribution of the impedance were in good agreement with the experiment, indicating the viability of the model. In particular, the R^2^ of the fits was always better than 0.98, and the relative error of estimate of all parameters varied during fitting, but did not exceed 10%. As the number of parameters to fit each impedance spectrum was significant, sensitivity analysis was carried out to assess the relevance of each parameter and its influence in the different regions of the frequency range studied [21]. For the purpose, every individual parameter was given sequential values 10% lower and 10% higher than the values that were returned as optimal by the fitting procedure, and the corresponding impedance spectra were calculated. As a result, it can be stated that all the studied parameters had a noticeable effect on the phase angle curves, and their values can be considered reliable. The effect of water chemistry on parameter estimates is discussed in the following section.

### 4.4. Effect of Water Chemistry on Kinetic and Transport Parameters

**Figure 9:**
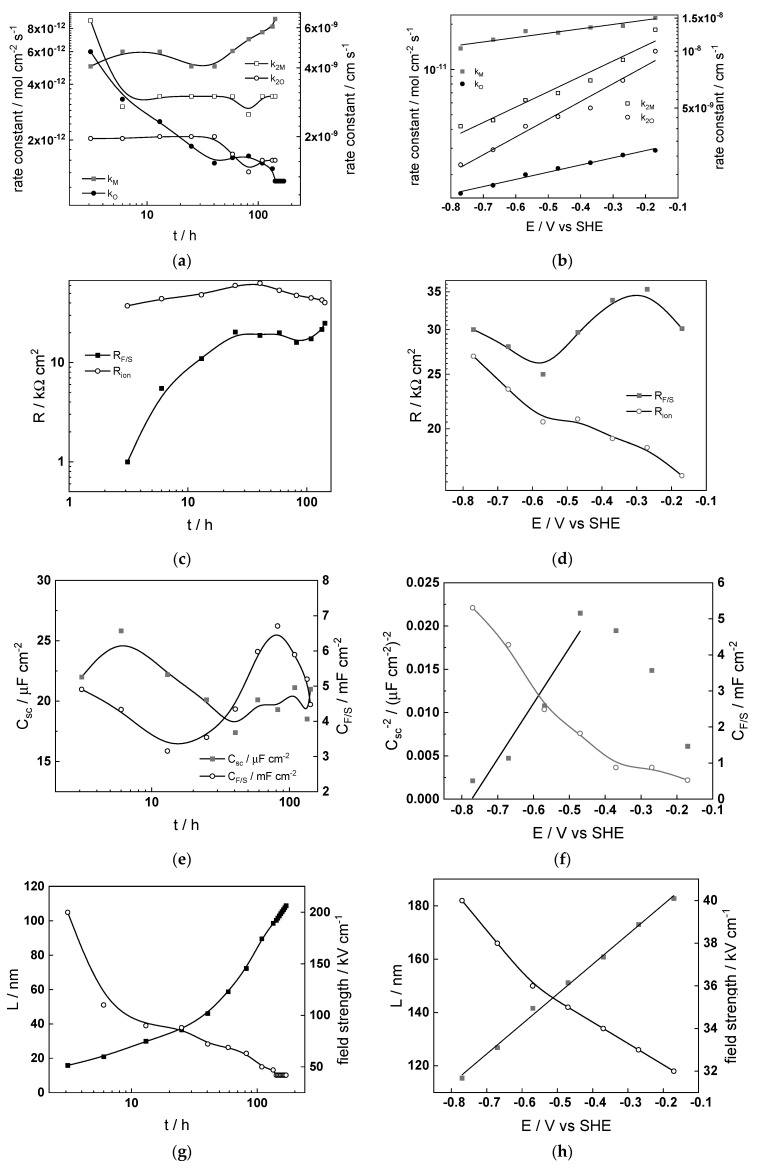
Kinetic and transport parameters in WWER BOC vs. time (**a**,**c**,**e**,**g**) and potential (**b**,**d**,**f**,**h**): *k*_1*O*_, *k*_1*M*_, *k*_2*O*_, and *k*_2*M*_ (**a**,**b**); R_F/S_ and *R_ion_* (**c**,**d**); *C_sc_* and *C_F/S_* (**e**,**f**); and L and field strength (**g**,**h**).

**Figure 10:**
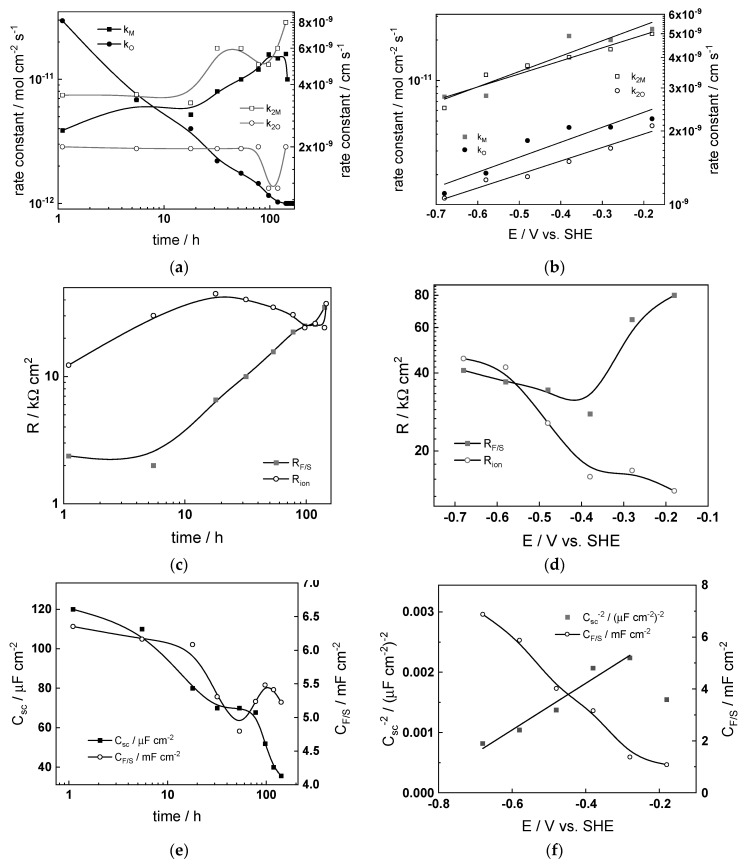

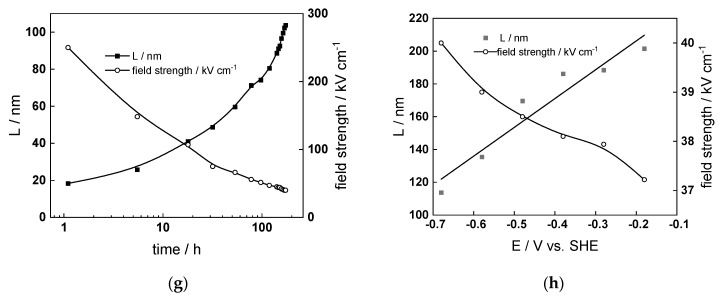
Kinetic and transport parameters in WWER MOC vs. time (**a**,**c**,**e**,**g**) and potential (**b**,**d**,**f**,**h**): *k*_1*O*_, *k*_1*M*_, *k*_2*O*_, and *k*_2*M*_ (**a**,**b**); *R_F/S_* and *R_ion_* (**c**,**d**); *C_sc_* and *C_F/S_* (**e**,**f**); and L and field strength (**g**,**h**).

**Figure 11:**
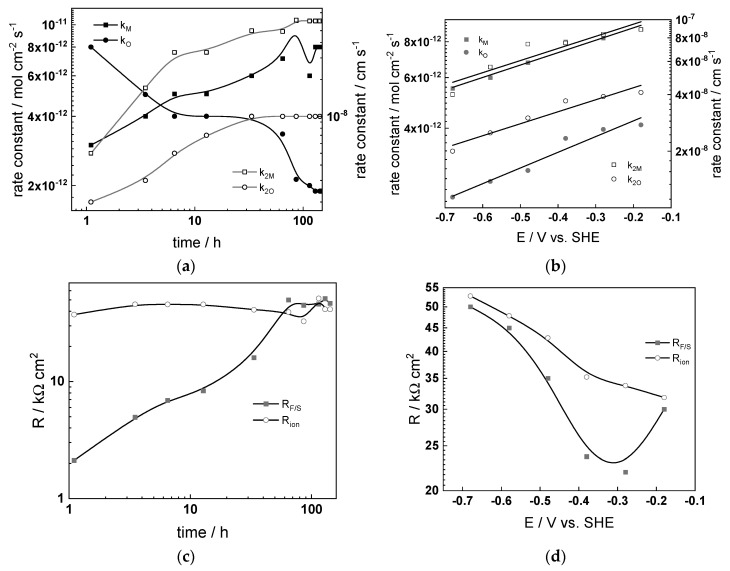

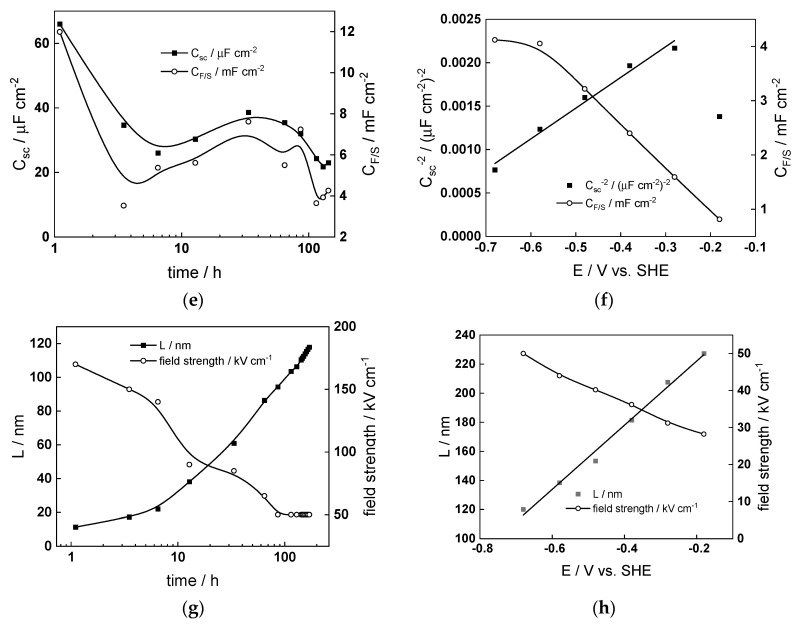
Kinetic and transport parameters in WWER EOC vs. time (**a**,**c**,**e**,**g**) and potential (**b**,**d**,**f**,**h**): *k*_1*O*_, *k*_1*M*_, *k*_2*O*_, and *k*_2*M*_ (**a**,**b**); *R_F/S_* and *R_ion_* (**c**,**d**); *C_sc_* and *C_F/S_* (**e**,**f**); and L and field strength (**g**,**h**).

**Figure 12:**
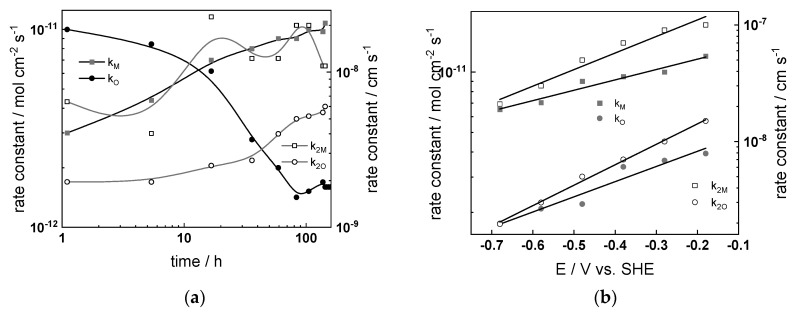

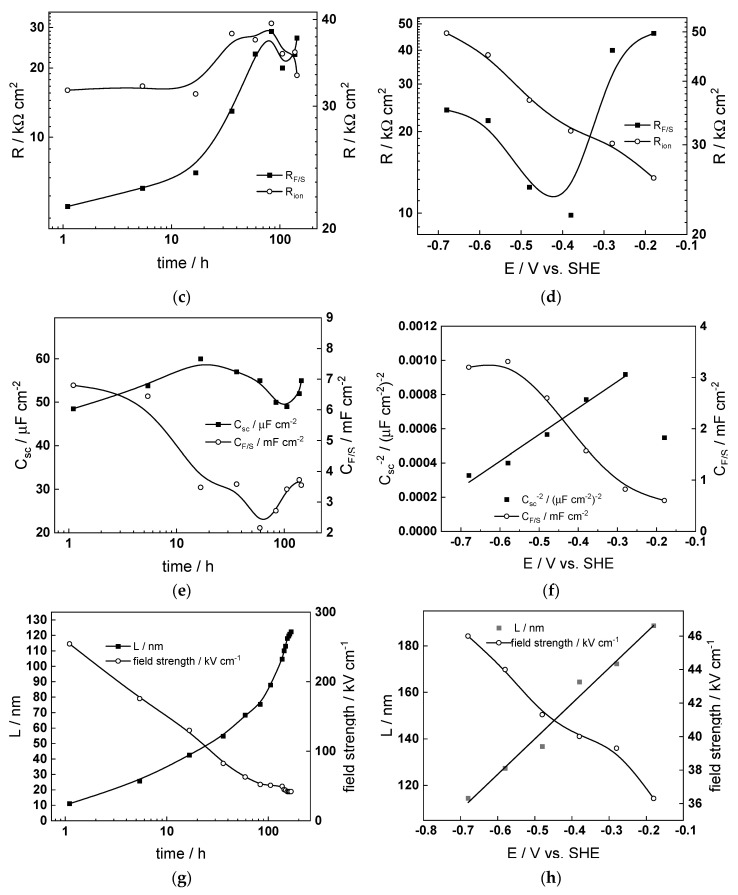
Kinetic and transport parameters in nominal PWR vs. time (**a**,**c**,**e**,**g**) and potential (**b**,**d**,**f**,**h**): *k*_1*O*_, *k*_1*M*_, *k*_2*O*_, and *k*_2*M*_ (**a**,**b**); *R_F/S_* and *R_ion_* (**c**,**d**); *C_sc_* and *C_F/S_* (**e**,**f**); and L and field strength (**g**,**h**).

The dependences of the estimated kinetic and transport parameters on exposure time and applied potential are collected in Figure 9, Figure 10, Figure 11 and Figure 12 for all the studied water chemistries. The resistance of ionic transport at zero frequency, expressed as

(8) $$R_{ion}=\frac{RT}{8F^{2}(1-\alpha )\left(k_{1O}+k_{1M}\right)}$$

is also plotted in the figures for comparative purposes. The parameters that were found to be independent of both time and applied potential are listed in Table 4.

The following conclusions on the effect of water chemistry can be drawn based on the parameters’ values:

- The differences between parameters estimated after exposure to the studied water chemistries were not very large, indicating that the corrosion and oxidation rates were rather similar regardless of the stage of operation (beginning, middle, or end of cycle). Most of the kinetic parameters stabilized after 40–50 h of exposure.
- The rate constants of oxidation at the steel/inner layer interface decreased with the duration of exposure, whereas the corresponding constants for metal oxidation with interstitial cation formation slightly increased. On the other hand, the rate constants of filling of oxygen vacancies and ejection of interstitial cations at the inner layer/coolant interface were quasi-independent on time, indicating no significant evolution of the energetics of this interface with exposure. This observation agrees with the very small evolution of both the space charge and interfacial capacitances with time after 40–50 h of exposure. It is worth noting that the values of C_F/S_ were rather large (several mF cm^−2^), i.e., it could be identified with a pseudo-capacitance of an adsorbed intermediate (for example, atomic H formed during reduction of water).
- All the rate constants obeyed, to a first approximation, an exponential dependence on applied potential in accordance with the model’s assumptions. The respective transfer coefficients estimated from the plots were rather low (of the order of 0.1–0.2), which is typical for systems with passive oxide films, indicating the similar energetics of the initial and transition states [22].
- The interfacial capacitance decreased with increasing potential, indicating a change in the nature of the adsorbed intermediate from atomic H to, e.g., adsorbed Cr(VI) species formed via transpassive oxidation. The space charge capacitance plotted vs. potential in Mott–Schottky coordinates (*C_sc_*^−2^ vs. E) indicated a change in the semiconductor type at around −0.4–−0.3 V, which could also be related to transpassive oxidation.

The comparison between the values of R_F/S_ and R_ion_ depending on time indicated that processes of oxidation and corrosion release were limited by both interfacial and bulk transport processes, especially at the end of exposure. Under anodic polarization, the transport processes gradually became faster than the charge transfer processes, being the least pronounced after exposure to nominal PWR coolant. The R_F/S_ parameter exhibited minima in the potential interval of −0.5–−0.4 V, which roughly coincides with the maxima in current–potential curves (Figure 3). As discussed above, this could be related to transpassive oxidation of Cr in the oxide. As a result of this oxidation, the protective ability of the oxide most probably decreased, since the ionic resistance decreased with increasing potential.

## 5. Conclusions

- Oxidation and corrosion of AISI 316L in simulated primary WWER coolant at different stages of operation in comparison to nominal PWR coolant were successfully characterized by a combination of in situ measurements and ex situ analytical techniques.
- In the present conditions, oxide growth and corrosion were found to be limited by both interfacial reactions and solid-state transport in the inner layer.
- Thin bi-layer oxides were formed on the steel after 168 h of exposure, with the inner layer tentatively identified as FeCr_2_O_4_ and the outer layer as FeNi_2_O_4_. An intermetallic Fe-Ni phase was also detected.
- The conduction mechanism in the steel/oxide/coolant system was successfully described by the mixed-conduction model. Kinetic and transport parameters, estimated as depending on exposure time and applied potential, indicated no significant differences between boron–potassium–lithium and boron–lithium primary water chemistries. Further investigations covering the effects of pH and temperature are needed in order to evaluate the general extent of the predictive ability of the modeling procedure. Concerning the industrial implications of the present study, it falls in line with findings from both EPRI and Framatome studies in demonstrating that a transition from Li− to (K + Li) primary water chemistry will not lead to enhanced uniform corrosion or oxide formation on reactor internals such as stainless steels. A companion study on the behavior of Alloy 690 in similar conditions is in progress and will be reported in the near future.

## Figures and Tables

**Table 1:** Composition of the studied material, as given by the supplier and analyzed with GDOES.

Weight %	C	Fe	Cr	Cu	Mn	Ni	P	S	Si	Mo
nominal	≤0.05	Bal.	17.0–18.0	≤0.30	1.2–2.0	12.0	≤0.035	≤0.02	≤0.8	2.4–2.7
analyzed	0.04	Bal.	17.6	0.30	1.3	12.0	0.03	0.02	0.76	2.6

**Table 2:** Thicknesses of the outer and inner layers of oxide, estimated by GDOES.

Coolant	Outer Layer/nm	Inner Layer/nm
WWER BOC	80 ± 5	110 ± 10
WWER MOC	70 ± 5	150 ± 12
WWER EOC	66 ± 5	144 ± 14
nominal PWR	74 ± 5	166 ± 14

**Table 3:** Identification of the phase composition of the oxide layer using data from grazing incidence XRD with appropriate standards: trevorite NiFe_2_O_4_ (8.334 Å), chromite FeCr_2_O_4_ (8.377–8.390 Å), FeNi_3_ (3.564 Å), and Fe_3_Ni_2_ (3.598 Å).

X-ray Angle of Incidence	Lattice Parameter of Spinel Phase/Å	Lattice Parameter of γ Phase/Å	Phase Composition
1°	8.350	3.595	Trevorite + γ phase
3°	8.377	3.601	Chromite + γ phase
8°	8.405	3.610	Chromite + γ phase

**Table 4:** Parameters that did not depend on time or applied potential.

Parameter	WWER BOC	WWER MOC	WWER EOC	PWR
10^8^ D_e_/cm^2^ s^−1^	0.50	2.0	2.0	7.0
10^17^ D_M/_cm^2^ s^−1^	0.50	0.60	0.50	0.50
10^17^ D_O/_cm^2^ s^−1^	0.30	0.35	0.30	0.30
α	0.85	0.85	0.85	0.85
α*_M_*	0.07	0.27	0.11	0.12
α*_O_*	0.11	0.27	0.13	0.18
α_2*M*_	0.11	0.07	0.08	0.18
α_2*O*_	0.12	0.08	0.08	0.22

## Data Availability

The data presented in this study are available upon request from the corresponding author (due to privacy).

## References

[1] McElrath J. (2014). Pressurized Water Reactor Primary Water Chemistry Guidelines.

[2] (2013). GAO-13-716. Managing Critical Isotopes: Stewardship of Lithium-7 Is Needed to Ensure a Stable Supply. https://www.gao.gov/products/gao-13-716.

[3] (2002). Review of VVER Primary Water Chemistry and the Potential for its Use in PWRs: Potassium Hydroxide and/or Ammonia Based Water Chemistries.

[4] (2015). Feasibility of Using Potassium Hydroxide for Primary Coolant pH Control in Pressurized Water Reactors.

[5] Fruzzetti K., Marks C., Reinders J., McElrath J., Wells D. Evaluation of Potassium Hydroxide for Reactor Coolant pHT Control in Western PWRs. Proceedings of the 20th International 15 Conference on Water Chemistry in Nuclear Reactor Systems.

[6] Chou P., Smith J., Demma A., Burke M., Fruzzetti K. Potassium Hydroxide for PWR Primary Coolant pH Control: Materials Qualification Testing. Proceedings of the 21st International Conference on Water Chemistry in Nuclear Reactor Systems.

[7] Fandrich J., Dudka E. Investigations of an Alternative Alkalization Strategy for Primary Coolant Conditioning of Pressurized Light Water Reactors. Proceedings of the 2018 Nuclear Power Plant Chemistry Conference (NPC 2018).

[8] Dingee J., Marks C., Fruzzetti K., McElrath J. Modeling Potassium Hydroxide for pH_T_ Control in Western-Style PWRs. Proceedings of the 21st NPC International Conference on Water Chemistry in Nuclear Reactor Systems.

[9] Chen K., Ickes M., Burke M., Was G. (2022). The effect of potassium hydroxide primary water chemistry on the IASCC behavior of 304 stainless steel. J. Nucl. Mater..

[10] Wang J., Zhu T., Bao Y., Liu X., Shi X., Guo X., Han Z., Andresen P., Zhang L., Chen K. (2023). Insights into the stress corrosion cracking propagation behavior of Alloy 690 and 316 L stainless steel in KOH versus LiOH oxygenated water. Corros. Sci..

[11] Kakitani K., Sugino W., Nakano Y., Sato K., Shimizu Y. (2023). Effect of KOH and dissolved hydrogen on oxide film and stress corrosion cracking susceptibility of Alloy X-750. Mech. Eng. J..

[12] Nigmatullina K., Rolph J., Cothran K., Ritchie J., Fruzzetti K., Perkins D., Chou P., Hussey D. Potassium Hydroxide for Western-Design PWRs: Plans for a Three-Cycle Monitored Campaign at TVA Sequoyah. Proceedings of the SFEN Conference on Nuclear Power Chemistry (NPC).

[13] Fruzzetti K., Marks C., Dingee J., Kim-Stevens K., Perkins D., Nigmatullina K. Potassium Hydroxide for Western-Design PWRs: Chemistry and Radiation Safety Assessments. Proceedings of the SFEN Conference on Nuclear Power Chemistry (NPC).

[14] Sinjlawi A., Dong L., Ickes M., Sun K., Was G. (2023). Irradiation assisted stress corrosion cracking of 347 stainless steel in simulated PWR primary water containing lithium hydroxide or potassium hydroxide. J. Nucl. Mater..

[15] Ferguson J., Arcis H., Tremaine P. (2019). Thermodynamics of Polyborates under Hydrothermal Conditions: Formation Constants and Limiting Conductivities of Triborate and Diborate. J. Chem. Eng. Data.

[16] Sharafutdinov R., Kharitonova N. (2011). The Problem of Optimizing the Water Chemistry Used in the Primary Coolant Circuit of a Nuclear Power Station Equipped with WWER Reactors under the Conditions of Longer Fuel Cycle Campaigns and Increased Capacity of Power Units. Therm. Eng..

[17] You C., Zabara M., Orazem M., Ulgut B. (2020). Application of the Kramers–Kronig Relations to Multi-Sine Electrochemical Impedance Measurements. J. Electrochem. Soc..

[18] Wan T., Saccoccio M., Chen C., Ciucci F. (2015). Influence of the Discretization Methods on the Distribution of Relaxation Times Deconvolution: Implementing Radial Basis Functions with DRTtools. Electrochim. Acta.

[19] Bojinov M., Kinnunen P., Lundgren K., Wikmark G. (2005). A Mixed-Conduction Model for the Oxidation of Stainless Steel in a High-Temperature Electrolyte—Estimation of Kinetic Parameters of Oxide Layer Growth and Restructuring. J. Electrochem. Soc..

[20] Que Z., Chang L., Saario T., Bojinov M. (2022). Localised electrochemical processes on laser powder bed fused 316L with various heat treatments in high-temperature water. Addit. Manuf..

[21] Betova I., Bojinov M., Kinnunen P., Lundgren K., Saario T. (2009). Influence of Zn on the oxide layer on AISI 316L(NG) stainless steel in simulated pressurised water reactor coolant. Electrochim. Acta.

[22] Sharifi-Asl S., Taylor M., Lu Z., Engelhardt G., Kursten B., Macdonald D.D. (2013). Modeling of the electrochemical impedance spectroscopic behavior of passive iron using a genetic algorithm approach. Electrochim. Acta.

